# The role of artificial intelligence in disease prediction: using ensemble model to predict disease mellitus

**DOI:** 10.3389/fmed.2024.1425305

**Published:** 2024-08-07

**Authors:** Qinyuan Du, Dongli Wang, Yimin Zhang

**Affiliations:** Key Laboratory of Traditional Chinese Medicine Classical Theory, Ministry of Education, Shandong University of Traditional Chinese Medicine, Jinan, China

**Keywords:** diabetes mellitus, disease prediction, machine learning, artificial intelligence, Stacking ensemble model

## Abstract

The traditional complications of diabetes are well known and continue to pose a considerable burden to millions of people with diabetes mellitus (DM). With the continuous accumulation of medical data and technological advances, artificial intelligence has shown great potential and advantages in the prediction, diagnosis, and treatment of DM. When DM is diagnosed, some subjective factors and diagnostic methods of doctors will have an impact on the diagnostic results, so the use of artificial intelligence for fast and effective early prediction of DM patients can provide decision-making support to doctors and give more accurate treatment services to patients in time, which is of great clinical medical significance and practical significance. In this paper, an adaptive Stacking ensemble model is proposed based on the theory of “error-ambiguity decomposition,” which can adaptively select the base classifiers from the pre-selected models. The adaptive Stacking ensemble model proposed in this paper is compared with KNN, SVM, RF, LR, DT, GBDT, XGBoost, LightGBM, CatBoost, MLP and traditional Stacking ensemble models. The results showed that the adaptive Stacking ensemble model achieved the best performance in five evaluation metrics: accuracy, precision, recall, F1 value and AUC value, which were 0.7559, 0.7286, 0.8132, 0.7686 and 0.8436. The model can effectively predict DM patients and provide a reference value for the screening and diagnosis of clinical DM.

## 1 Introduction

Diabetes mellitus (DM) is a metabolic disease clinically characterized by chronic hyperglycemia, dyslipidemia and protein abnormalities, and other symptoms that increase the risk of morbidity and mortality, of which type 2 diabetes mellitus (T2DM) is a major public health challenge globally, and the assessment and management of this chronic disease carries a heavy economic burden (1–3). Worldwide, 537 million adults (aged 20–79) have diabetes (10%), and this number is expected to rise to 643 million by 2030 and 783 million by 2045 (4, 5).

Along with the rapid development of the intersection of artificial intelligence and medical diagnostics, machine learning (ML) has once become the most concerned topic among researchers, which can provide accurate predictive analysis of diseases, effectively identify high-risk factors as well as patients with high morbidity, and then provide accurate decision support for hospital administrators (6). By mining potential healthcare data through machine learning and constructing a novel DM prediction model, early warning of high-risk groups can be performed, and appropriate healthcare management can be taken to patients in advance, which can also provide certain decision support to doctors and reduce the rate of missed diagnosis and misdiagnosis (7).

Initially, researchers predicted DM through traditional machine learning and verified that random forest (RF) based on tree model has better prediction effect (8–12). Some researchers focused on the preliminary data processing to get a better DM prediction model using feature selection and data imbalance processing (13–15). Meanwhile, considering the influence of different factors on diabetes, researchers began to study the three aspects of age, gender and geography, and obtained a better prediction effect of the targeted population prediction model (16–18).

In recent years researchers began to consider the use of ensemble learning to predict diabetes and obtained diabetes prediction models that are superior to traditional machine learning (19, 20). In DM prediction, although machine learning is superior to traditional statistical methods, most of the research has focused on a single prediction model. Each prediction model has its advantages, disadvantages and limitations, and researchers use ensemble learning to combine the advantages of a single prediction model to build a more powerful ensemble model, of which the most effective Stacking ensemble model is gradually applied (21–23). From the perspective of single classification model, using Stacking ensemble model can solve the limitations of single classification model, but the selection of base classifiers and meta-learners of Stacking ensemble model has randomness (24).

To solve the above-mentioned problem, this paper follows the theory of “error-ambiguity decomposition” (25) and designs an adaptive Stacking ensemble model, which can further improve the prediction performance of DM model.

## 2 Materials and methods

**FIGURE 1:**
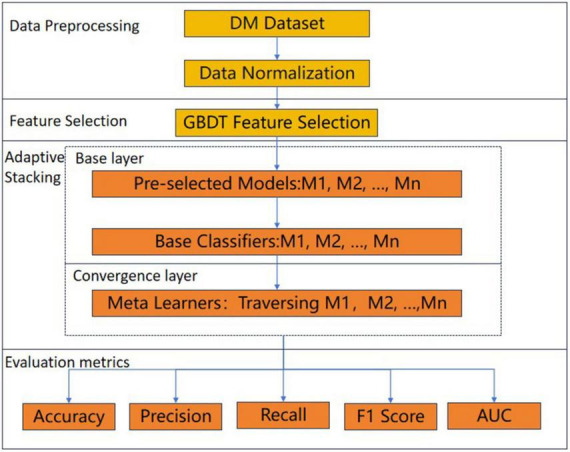
Prediction process flow chart.

In this paper, an adaptive Stacking ensemble model is designed and implemented on the DM dataset to predict DM patients, and the overall process framework is shown in Figure 1. Firstly, the DM dataset was normalized, followed by feature screening using the gradient boosting decision tree (GBDT) (26) feature selection method, and next the adaptive Stacking ensemble model was constructed by first adaptively selecting the base classifiers from *n* pre-selected models, and then traversing the models in the selection of meta-learners. The performance of the adaptive Stacking ensemble model, proposed in this paper was evaluated by five evaluation metrics: accuracy, precision, recall, F1 value and AUC value.

### 2.1 Dataset

The dataset selected for this paper is a balanced dataset processed on CDC’s BRFSS2015, which has the same proportion of diabetic and non-diabetic interviews, totaling 70,691 samples. This dataset contains 21 characteristic variables as shown in Table 1.

**TABLE 1:** CDC’s BRFSS2015.

ID	Feature	Detailed description
1	HighBP	High blood pressure
2	HighChol	High cholesterol
3	CholCheck	Had a cholesterol test within 5 years
4	BMI	Body mass index
5	Smoker	Smoker
6	Stroke	Stroke
7	HeartDiseaseor	Coronary heart disease or myocardial infarction
8	PhysActivity	Physical activity in the last 30 days
9	Fruits	Fruit 1 or more times per day
10	Veggies	Veggie 1 or more times per day
11	Alcoholic	Alcoholic
12	AnyHealthcare	Have any type of health insurance
13	NoDocbcCost	In the past 12 months, have you needed to see a doctor but were unable to do so due to cost?
14	GenHlth	Health status
15	MentHlth	Mental health status
16	PhysHlth	Health status
17	DiffWalk	Have severe difficulty walking or climbing stairs
18	Sex	Sex
19	Age	Age
20	Education	Educational level
21	Income	Income situation

### 2.2 Feature selection

Feature selection is an important technique in machine learning to filter out the most valuable and relevant sample features in the data for use in building machine learning models (27, 28). The purpose of feature selection is to reduce the number of sample features in the data, improve the accuracy and operational efficiency of the model, reduce the risk of overfitting, and improve the interpretability of the model (29). Not all sample features have a significant impact on the prediction results, which contains many sample features with low or irrelevant contribution to the prediction results, and too many sample features will cause computational resource consumption and reduce the training speed of the model, and may also reduce the accuracy of the model, so this chapter is to eliminate the sample features with low or irrelevant contribution to the prediction results. There are three common feature selection methods: filter, wrapper, and embedding (30). Since embedding methods have better predictive performance than filter methods and run much faster than wrapper methods (31), our study uses the embedding method GBDT to select feature variables.

GBDT is an ensemble learning method that improves the predictive performance of a model by constructing a series of weak learners (usually decision trees). The basic idea of GBDT is to combine multiple simple models (weak learners) so that each new model corrects as much as possible the errors of the previous model. Since the decision tree splits based on the importance of the features and features with high importance are more frequently selected as split points, GBDT can rank the importance of the features and handle high dimensional data. Secondly, GBDT can handle different types of features, including numerical and categorical. Finally, GBDT focuses on the residuals of the current model rather than modeling the target values directly, which makes the model more tolerant to noisy data.

When we use GBDT with embedding method to obtain feature importance ranking, one of the problems we face is the inability to accurately interpret the impact of individual features on the final prediction results. To solve this problem, we used a technique called feature interpolation method (32). This method represents the explanatory model as a linear function of the feature interpolation values, thus providing a clearer understanding of the model behavior. By this method we can reduce the repetition rate and better understand the contribution of individual features to the prediction results. The method is formulated as follows.


(1) $$l\left(z^{\prime }\;=\;\emptyset_{0}+\sum_{i=1}^{N}\emptyset_{i}Z_{i}^{\prime }\right)$$

Where *N* is the number of features, ∅_*i*_ is the value of the feature attribute of the feature, $Z_{i}^{\prime }=0$ or 1 to indicate whether the feature is observed or not, where the feature attribute can be regarded as the “feature contribution.”

In order to compute the ∅_*i*_ values in Equation 1, a tree-value estimation method based on game theoretic ideas (33), the SHAP method, is introduced as feature attribute values. In this method, the model *f* and the set *S* contain non-zero indexes in *z*′ and each feature has the classical Shapley value attribute ∅_*i*_, which is formulated in Equation 2.


(2) $$\emptyset_{i}=\sum_{S\in M\,\left\{i\right\}}\frac{\left|S\right|!\,\left(N-\left|S\right|-1\right)!}{N!}\,\left[f\,\left(S\cup \left\{i\right\}-f\,\left(S\right)\right)\right]$$

where *M* is the set of all input features.

The SHAP method is a locally accurate, personalized feature attribution method that, unlike tree model gain, provides consistent global feature attribute results (34). In our study, we use the SHAP method for feature filtering and interpretation of individual feature attributes, which helps to reduce the repetition rate and provide a clearer understanding of the impact of each feature on the results.

### 2.3 Model building

When the base classifiers predict accurately, the greater the variability of the base classifiers, the better the integration of the model will be, which is the famous “error- ambiguity decomposition” theory. This implies that the variability of the base classifiers should be taken into account while guaranteeing the prediction effect of the base classifiers.

**FIGURE 2:**
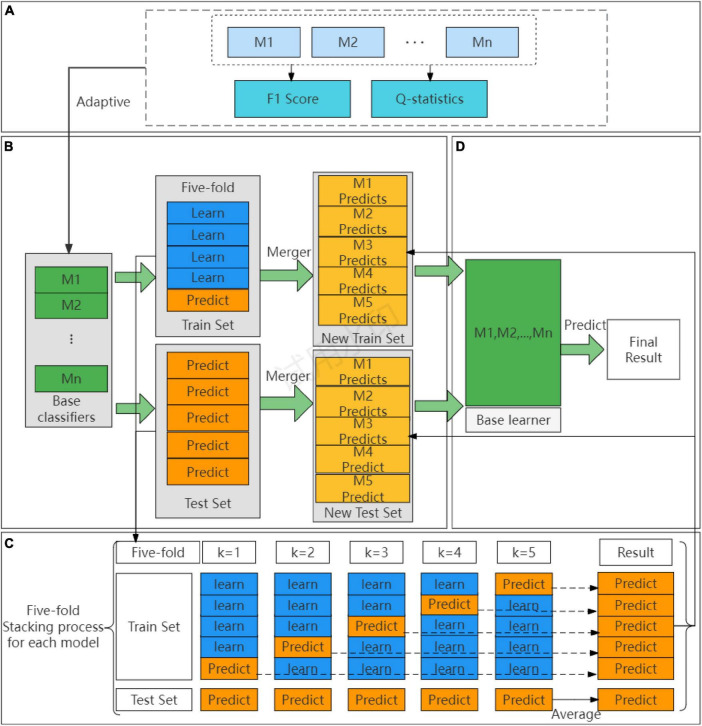
The process of adaptive Stacking model. **(A)** Adaptive selection base classifier process. **(B)** Process of building a base classifier. **(C)** Cross-validation detailed process. **(D)** Building a meta-learner process.

In principle, as long as the base classifier of the Stacking ensemble model predicts well (35), the number of layers of the Stacking ensemble model can be stacked infinitely, but this increases the complexity of the model. Therefore, we try to reduce the model complexity as much as possible while ensuring the prediction effect of the model, and only select the Stacking ensemble model, with the two-layer structure of the base layer and the convergence layer. To solve the problem of randomness of the traditional Stacking ensemble model, in selecting base classifiers and meta-learners, this paper proposes an adaptive Stacking ensemble model, and the process is shown in Figure 2.

The first step is to adaptively construct the base classifier of the Stacking base layer, as shown in Figure 2A. When choosing the base classifiers, the traditional Stacking ensemble model usually select the classifiers with good prediction effect, ignoring the principle of “error- ambiguity decomposition.” In this paper, we design a method to adaptively construct the base classifiers of Stacking, from the pre-selected models of *M*1,*M*2,…,*Mn*, according to the F1 value of the comprehensive evaluation metrics, we set a threshold to adaptively select the pre-selected models from the high to the low to construct the base classifiers, which is a step to ensure the prediction effect of the models. To ensure the variability of the models, this paper chooses the Q-statistics method (36) to compare the variability between the pre-selected models.

The detailed steps of the Q-statistics method are as follows, labeling the DM dataset as *Z* = *z*_1_,*z*_2_,…,*z*_*n*_, the pre-selected model as *M*_*i*_, after using *n* pre-selected models for classification prediction, if the pre-selected model *M*_*i*_ predicts the ensemble correctly it will be 1, and the prediction is wrong it will be 0, and the relationship between the two pre-selected models is shown in Table 2.

**TABLE 2:** Relationship between two pre-selected model.

	*M*_*k*_ correct (1)	*M*_*k*_ wrong (0)
*M_i_* correct (1)	*N*_11_	*N*_10_
*M_i_* wrong (0)	*N*_01_	*N*_00_

The differential Q-statistics for the two pre-selected model *M*_*i*_,*M*_*k*_ are shown in Equation 3 as follows.


(3) $$Q_{i,k}=\frac{N_{11}\,N_{00}-N_{01}\,N_{10}}{N_{11}\,N_{00}+N_{01}\,N_{10}}$$

Statistically, the expected value of two completely independent pre-selection models *Q*_*i,k*_ is 0. The range of *Q* is between [−1, 1], and the smaller the absolute value, the greater the variability between the pre-selection models. In the L pre-selection models of this paper, the average value of *Q* is shown in Equation 4.


(4) $$Q_{a\,v\,g}=\frac{2}{L\,\left(L-1\right)}\,\sum_{L-1}^{i=1}\sum_{L}^{k=i+1}Q_{i,k}$$

The steps for adaptively constructing a base classifier for the Stacking ensemble model, are shown below:

(1) Train *n* pre-selected models *M*_1_,*M*_2_,,*M*_*n*_.
(2) Calculate the comprehensive evaluation metrics F1 value of the *n* pre-selected models, and set the threshold as the average F1 value.
(3) Eliminate the models with F1 values smaller than the threshold and retain the models with F1 values larger than the threshold.
(4) Models with small variance are eliminated and models with large variance are retained based on Q-statistics.
(5) Select *M*_1_,*M*_2_,,*M*_*n*_ as the final base classification.

Figure 2B shows the cross-validation part of the adaptive Stacking ensemble model. After adaptive selection as base classifiers, the train set and test set are divided according to an 8:2 ratio. In the train set, each base classifier using five-fold cross-validation. Taking five base classifiers *M*1,*M*2,,*M*_5_ as an example, the specific operation is shown in Figure 2D. A base classifier can get five predictions, which are vertically stacked into a one-dimensional matrix. Five base classifiers can be combined into a five-dimensional matrix as a new train set for the convergence layer. In the test set, again each base classifier using five-fold cross-validation and again five predictions are obtained. To ensure the division ratio between the train set and test set, the predictions of the test set are horizontally averaged to obtain a one-dimensional matrix. The predictions of the five base classifiers are combined into a five-dimensional matrix that serves as a new test set for the convergence layer.

The second step adaptively constructs the meta-learner of the Stacking ensemble model. As shown in Figure 2C, this paper traverses the whole base classifier model to select the meta-learner, and obtains the final prediction result through the meta-learner.

Finally, to better understand the implementation process of the Adaptive Stacking algorithm, this paper gives the pseudo-code of the Adaptive Stacking algorithm, as shown in Algorithm 1.

**Algorithm 1:** Adaptive Stacking algorithm.

Symbols are defined: threshold λ, *F*_*i*_*^N^* is the F1 value of *M*_*i*_, ⊙ is the model satisfying the discreteness condition, *M* is the set of base classifiers, the train set *T* and the test set *S, T_*ft*_* is the training set for the five-fold cross-validation, *S*_*fv*_ is the test set for the five-fold cross validation, *εl* is the base classifier, *LR*_*meta*_ is the meta learners, *T^l^* is the train set for *εl*, and *S^l^* is the test set for *εl*
1. **For** *N* = [M_1_, M_2_,…,M_*n*_] // Preselection model
2. **If** *F_*i*_*^N^* >λ* // Determine if the F1 value of model *M*_*i*_ is greater than a threshold value
3. **If** *M* = Ø // Determine if *M* is empty
4. *M* = *M + M_*i*_ //* Model *M*_*i*_ is added to *M*
5. **If** *M*_*i*_ ⊙ *Mj //* Determine whether two models satisfy the difference condition
6. *M = M + Mj* // Model *M*_*j*_ is added to *M*
7. **For** *l* = M = [M_1_, M_2_, …, M_*n*_] // Base classifiers
8. **For** k = 1,2, …,5
9. *T_*ft*_*^l^* →⊙l //* Use *T_ft_^l^* to train *εl*
10. *S_*fv*_*^l^*→εl → train*_*f*_, *S*^l^*→εl → test_*f*_ //* Predict *S_fv_^l^,S^l^* by *εl* to get *train_f_,test_f_*
11. *train_*l*_ = (train*_1_ + *train*_2_ + … + *train*_5_) // Vertical stack
12. *test*_*l*_ = (*test*_1_ + *test*_2_ + …+ *test*_5_)/5 // Level average
13. *train_*new*_* = [*train*_1_*,train*_2_*,… train*_5_*]* and *train_*new*_* = [*train*_1_*,train*_2_*,… train*_5_]
14. *train_*new*_ → LR_*meta*_* //Train *LR*_*meta*_ with *train*_*new*_
15. *test*_*new*_ → *LR*_*meta*_ → *result*_*pre*_ //Predict *test*_*new*_. with *LR*_*meta*_ to get the final result
16. **Return** *result_*pre*_ //* Returns the final prediction

Typically, machine learning evaluates the performance of a model using True Positive (TP), True Negative (TN), False Positive (FP) and False Negative (FN) metrics. The commonly used accuracy, precision, recall and F1 score are calculated from these metrics, which can be calculated by referring to Equations 5–8.


(5) $$A\,c\,c\,u\,r\,a\,c\,y=\frac{T\,P+T\,N}{T\,P+T\,N+F\,P+F\,N}$$

(6) $$P\,r\,e\,c\,i\,s\,i\,o\,n=\frac{T\,P}{T\,P+F\,P}$$

(7) $$R\,e\,c\,a\,l\,l=\frac{T\,P}{T\,P+F\,N}$$

(8) $$F\,1=2\times \frac{P\,r\,e\,c\,i\,s\,i\,o\,n\times R\,e\,c\,a\,l\,l}{P\,r\,e\,c\,i\,s\,i\,o\,n\times R\,e\,c\,a\,l\,l}$$

## 3 Results and discussion

### 3.1 Results of feature selection and analysis

**FIGURE 3:**
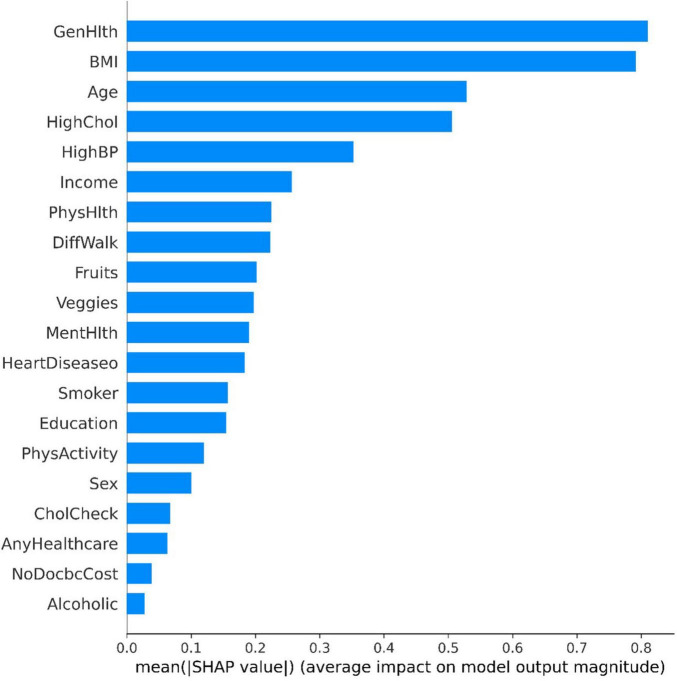
Global feature contributions.

The tree SHAP method calculates the individual contribution value of each feature in the sample dataset. Figure 3 demonstrates the global feature contribution in the GBDT model, the horizontal coordinate represents the sample feature contribution, the larger the value, the more important the sample feature is, and the vertical coordinate is the sample feature based on the feature importance from the largest to the smallest. From the figure, it can be seen that the features “GenHlth” and “BMI” have significant contribution degrees, which indicates a strong correlation with diabetes. While the inverse features “CHolCheck,” “AnyHealthcare,” “NoDocbcCost,” and “Alcoholic” features have less than 0.1 contribution, this paper directly excludes these four features and finally retains 16 sample features.

**FIGURE 4:**
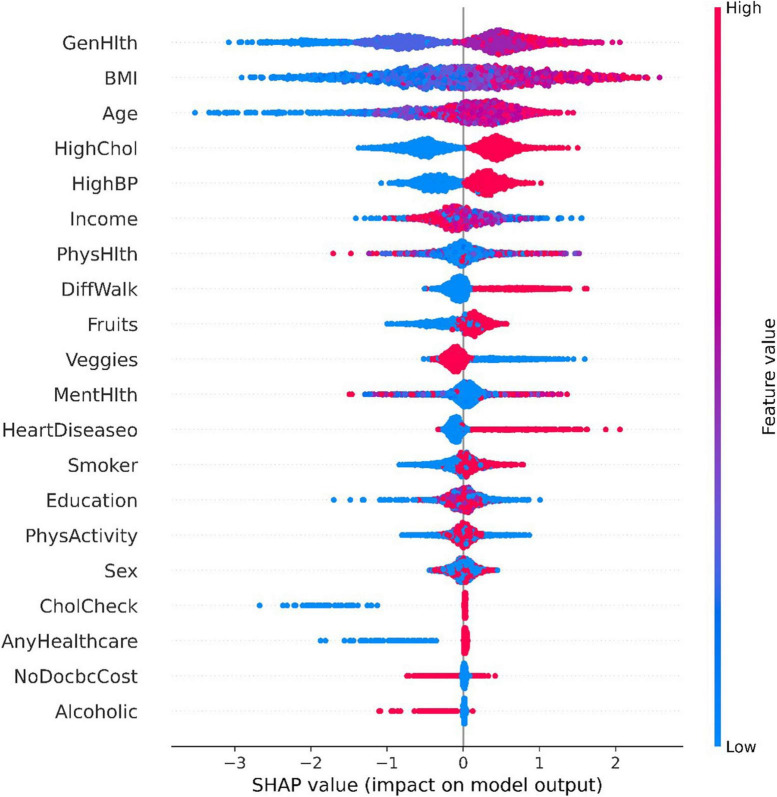
Comparision of individual feature contributions.

The Tree SHAP values depend on how the features are interpreted, so we can obtain the feature interpretation for each sample from the model (37). Figure 4 presents some information about the contribution of individual features to the model output and details how their values affect the model. the *x*-axis indicates the magnitude of the feature contribution, while the magnitude of the feature value is indicated by the color of the different points. The highest contribution of characteristics, “GenHlth” indicates that the poorer the physical condition the more likely to get diabetes, and similarly from the second and third ranked “BMI” and “Age,” it can be seen that the higher the weight coefficient the more likely to get diabetes, and the higher the age the more likely to get diabetes.

### 3.2 Results of the proposed adaptive Stacking ensemble model

**FIGURE 5:**
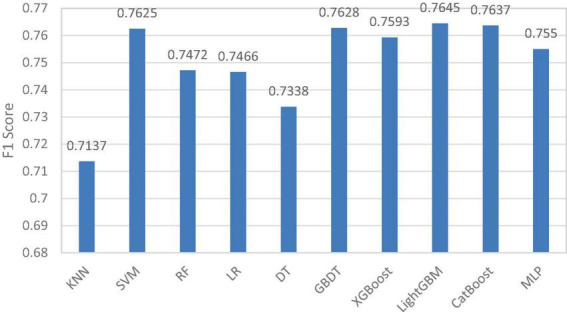
F1 values for 10 pre-selected models.

In the adaptive Stacking ensemble model, the 10 pre-selected models are sorted according to the size of the F1 value, and the sorting results are shown in Figure 5. The horizontal coordinates in the figure indicate the 10 pre-selected models and the vertical coordinates indicate the F1 values of the pre-selected models.

In this paper, we set the average of F1 values as the threshold and first excluded four pre-selected models, K-nearest neighbor (KNN), RF, logistic regression (LR) and decision tree (DT). Four models, support vector machine (SVM), multilayer perceptron (MLP) and XGBoost and CatBoost, are selected as base classifiers from the remaining six pre-selected models adaptively based on Q-statistics. The top-ranked GBDT model and LightGBM model have better prediction results, but the difference between them and CatBoost model is proved to be small by Q-statistics method, so these two models are eliminated in adaptive way. In the selection of meta-learner, this paper traverses all the base classifier models and selects the optimal meta-learner according to the evaluation metrics.

In this paper, the prediction results of 10 pre-selected models, traditional Stacking ensemble model, and the adaptive Stacking ensemble model proposed in this paper are compared by four evaluation metrics: Accuracy, Precision, Recall and F1 value, and the comparison results are shown in Table 3. It can be seen that compared with the traditional machine learning models KNN and SVM, the traditional Stacking ensemble model outperforms the single classification model in predicting DM. The adaptive Stacking ensemble model proposed in this paper has very high accuracy, recall and F1 values of 0.7559, 0.8132 and 0.7668, which are higher than the traditional Stacking ensemble model. The adaptive Stacking ensemble model proposed in this paper is completely better than the traditional Stacking ensemble model, and can adaptively make the best adjustment. This shows that the adaptive Stacking ensemble model has obvious advantages in predicting DM.

**TABLE 3:** Results of classifiers.

Model	Accuracy	Precision	Recall	F1 score
KNN	0.7068	0.6954	0.733	0.7137
SVM	0.74.9	0.7217	0.8081	0.7625
RF	0.7371	0.7176	0.7794	0.7472
LR	0.741	0.7289	0.7652	0.7466
DT	0.7232	0.7048	0.7652	0.7338
GBDT	0.7536	0.73.3	0.7949	0.7628
XGBoost	0.7493	0.7283	0.7932	0.7593
LightGBM	0.7547	0.7331	0.7987	0.7645
CatBoost	0.7557	0.7336	0.8008	0.7637
MLP	0.7493	0.7362	0.7746	0.755
Stacking	0.7554	0.7307	0.8007	0.7668
Adaptive Stacking	**0.7559**	0.7286	**0.8132**	**0.7686**

The results with the best predictions are highlighted in bold.

**FIGURE 6:**
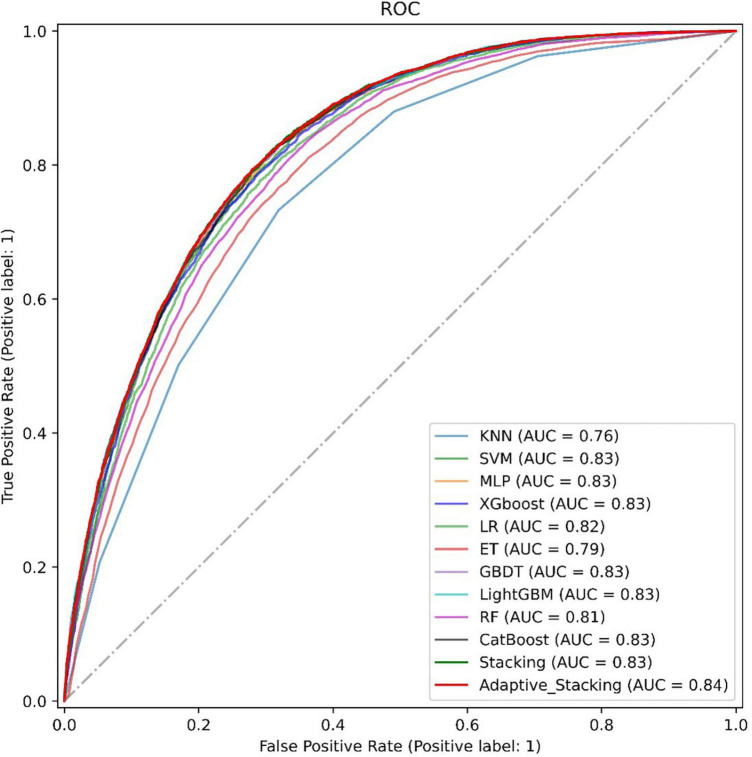
ROC curve on DM.

Finally, this paper plots the ROC curves of 11 representative models with the adaptive Stacking ensemble model, as shown in Figure 6. In the dataset of this paper, the ensemble learning model predicts better than traditional machine learning, and the AUC value of the ensemble learning model reaches 0.83. Among the Stacking ensemble model is better than the Bagging model and the Boosting model. The AUC value of the adaptive Stacking ensemble model reaches 0.84, which is higher than the traditional Stacking ensemble model. This shows that the adaptive superposition ensemble model proposed in this paper has excellent prediction effect in predicting DM.

## 4 Conclusion

In this paper, we propose a DM prediction model based on adaptive Stacking and analyze it in comparison with 10 pre-selected models and traditional Stacking ensemble model by five evaluation metrics: accuracy, precision, recall, F1 value and AUC value. The results show that the adaptive Stacking ensemble model proposed in this paper outperforms other models in several evaluation metrics, with accuracy, precision, recall, F1 value, and AUC value of 0.7559, 0.7268, 0.8132, 0.7686, and 0.8436, which suggests that the adaptive Stacking ensemble model, proposed in this paper is able to integrate the advantages of a single model and adaptive selection of pre-selected models to obtain better prediction results, which can provide clinical diagnostic advice and decision support for doctors and provide patients with appropriate medical and health management as early as possible. Although the adaptive Stacking model proposed in this study has a better prediction effect compared to a single model, the model complexity is high, and the complexity of the model needs to be further optimized according to the actual application scenarios. In addition, this study is limited to the study of machine learning models, and will collect more datasets and try to use deep learning models for the study. Finally, we stay on top of recently released healthcare policies, continually communicating with local hospitals and healthcare professionals to collect data on patient metrics.

## Data availability statement

Publicly available datasets were analyzed in this study. This data can be found here: https://www.kaggle.com/datasets/alexteboul/diabetes-health-indicators-dataset.

## Author contributions

QD: Data curation, Software, Writing – original draft, Writing – review & editing. DW: Data curation, Supervision, Formal analysis, Project administration, Writing – review & editing. YZ: Writing – original draft, Writing – review & editing.
